# Very-Large-Scale Integration-Friendly Method for Vital Activity Detection with Frequency-Modulated Continuous Wave Radars

**DOI:** 10.3390/s25072151

**Published:** 2025-03-28

**Authors:** Krzysztof Ślot, Piotr Łuczak, Paweł Kapusta, Sławomir Hausman, Arto Rantala, Jacek Flak

**Affiliations:** 1 Institute of Applied Computer Science, Lodz University of Technology, Stefanowskiego 18, 90-537 Łódź, Poland; krzysztof.slot@p.lodz.pl (K.Ś.); pawel.kapusta@p.lodz.pl (P.K.); 2 Institute of Electronics, Lodz University of Technology, Aleje Politechniki 10, 90-537 Łódź, Poland; slawomir.hausman@p.lodz.pl; 3 VTT Technical Research Centre of Finland Ltd., P.O. Box 1000, FI-02044 Espoo, Finland; arto.rantala@vtt.fi (A.R.); jacek.flak@vtt.fi (J.F.)

**Keywords:** recurrent neural networks, analog VLSI circuits, vital activity detection, FMCW radar

## Abstract

A simple algorithm for respiratory activity detection in data produced by Frequency-Modulated Continuous-Wave (FMCW) radars is presented in this paper. The proposed computational architecture can be directly mapped onto custom digital–analog VLSI hardware, which is a unique approach in research on intelligent FMCW sensor development, offering a potential energy-efficient data analysis solution for target applications, such as preventing human trafficking or providing life-sign detection under limited visibility. The algorithm comprises two main modules. The first one summarizes radar-produced data into a descriptor reflecting the amount of motion that occurs within appropriately determined time intervals. The second one classifies a sequence of the produced descriptors using a recurrent neural network composed of gated recurrent units. To ensure the algorithm’s implementation feasibility, an analog VLSI circuit comprising its main functional blocks has been designed, manufactured, and tested, providing constraints for neural model derivation. The adverse effects of the primary constraint, the severe restriction on admissible weight resolution, have been handled by introducing a novel training loss component and a simple mechanism for diversifying the effective weight sets of different network neurons. Experimental evaluation of the presented method, performed using the dataset of indoor recordings, indicates that the proposed simple, hardware implementation-friendly algorithm provides over 94% human detection accuracy and similar F1 scores.

## 1. Introduction

Remote monitoring of basic vital parameters, such as breathing or heart rate, is one of the key components of emerging health monitoring infrastructure in the technology-assisted living paradigm [1]. In addition to the basic requirements that need to be fulfilled by candidate vital activity monitoring systems, such as measurement accuracy and reliability, several other issues are also important, including unobtrusive data acquisition or preservation of subjects’ privacy. A possible technology that can be considered in developing vital activity systems that satisfies these additional requirements is a Frequency-Modulated Continuous-Wave (FMCW) radar. As FMCW radars deliver only basic information on the location and motion of objects present within radar-monitored spaces, it is challenging to determine the identity of observed subjects. Motion tracking ability, offered by FMCW radars, can be used for vital activity monitoring and other essential applications, such as the more generic one, vital activity detection, which enables, e.g., searching for survivors under severely limited visibility or preventing human trafficking in border control inspection.

The use of FMCW radars for vital activity monitoring is an ongoing research field, and several remarkable contributions, often stimulated by progress in deep learning, have been proposed so far. These include, for example, breathing rate (BR) estimation for single [2,3,4,5] or multiple [6] subjects, heart rate estimation [4,7,8,9], or activity recognition based on motion patterns retrieved from FMCW radar data [10]. Radar-based contactless breathing rate measurements have also found use in clinical research particularly related to sleep apnea detection [11]. The common denominator of these achievements is the reliance on discrete fourier transform-based signal preprocessing algorithms and the application of deep neural data analysis models.

The main difficulty in using FMCW radars for the evaluation and tracking of small object displacements is the so-called phase-wrapping problem. If the magnitude of an object’s displacement exceeds the radar-emitted wavelength, the linear relation between distances and the corresponding phase shifts used for its measurement no longer hold: phase jumps (from 2π to 0 or vice versa) transform the linear displacement-shift mapping into a periodic sawtooth function. This phenomenon is especially eminent in the case of BR monitoring, where centimeter-range breathing-related chest displacements, combined with intentional or spontaneous motion activity, are confronted with millimeter-range radar wavelengths.

As FMCW-based health monitoring systems can be considered intelligent edge devices, one of the key requirements for their design is the minimization of energy consumption. The default implementation for the majority of the proposed algorithms is a general-purpose CPU- or GPU-based computational architecture, where the aforementioned requirement is violated, and only a handful of solutions target energy-efficient, primarily digital, custom computational architectures [12,13]. Mapping of the data processing pipeline directly onto the hardware enables the optimization of its execution, for example, using an energy-efficiency criterion. This particular perspective, combined with the heavy reliance of the data analysis algorithms on neural computing, brings attention to the potential analog VLSI implementation technology. Analog computing offers the most energy-efficient realization of neural computations, which are performed by exploiting the physical properties of circuit elements. Therefore, the development of custom, application-specific architectures that combine analog computing with digital control retains popularity as an important research direction.

The objective of the present research was to develop a simple yet reliable breathing activity detection method that exploits FMCW radar-generated data and is suitable for their physical implementation in the form of a low-power standalone edge device. The proposed computational architecture involves two distinct modules targeting digital and analog hardware. The first step of the data analysis pipeline—feature extraction, which is intended for the digital implementation—produces a sequence of simple descriptors of information comprised of the FMCW radar scans. The second step, sequence classification, intended for its analog implementation, involves a recurrent neural network. To attain the stated objective, we sought ways to minimize the complexity of both of the considered data analysis phases and proposed to summarize the information on monitored processes using an easy-to-compute differential descriptor of radar-produced data and a minimalistic neural model based on gated recurrent units [14,15].

The formulated algorithm and its underlying computational architectures have been verified using a custom dataset, including over seven hours of indoor recordings taken with or without the presence of humans, acquired using the IWR1443BOOST radar device. To provide solid foundations for simulating the algorithm execution, an analog VLSI circuit comprising the main neural components has been designed, manufactured, and tested. The measurements delivered the key design constraints for the development of feasible neural architectures. Experimental evaluation results show that the proposed method is capable of attaining from 92% to 94.1% accuracy and similar F1 scores for several frugal analog VLSI-feasible neural models comprising only between one and two thousand parameters.

The two main novel contributions of this paper are the proposal of a simple descriptor for summarizing motion-related information provided by FMCW radars and a strategy for adapting neural models for their analog hardware implementation. This strategy involves the application of the novel quantization-loss component and a simple mechanism enabling the effective diversification of the heavily quantized individual neuron weight vectors.

### Related Work

Two main research areas are ancillary to the considered task: algorithmic concepts proposed for remote, FMCW radar-based vital activity detection, and the development of energy-efficient hardware for implementing these algorithms.

The FMCW technique can be dated back to the 1960s as a concept of linear frequency modulation (LFM) or “chirp” signals, which are critical to FMCW radar systems [16,17]. Information delivered by FMCW radar devices is embedded in mixtures of intermediate-frequency (IF) components of in-phase and quadrature signals. IF component frequencies are proportional to distances of wave-reflecting surfaces (where the scaling factor is determined by the slope of the adopted radar frequency modulation), whereas the phase evolution Δϕ correlates with the target displacements Δd and the carrier frequency *f* using the linear relation Δϕ=4πfΔd/c, where *c* is the speed of the electromagnetic wave.

For typical carrier-wave frequencies used in current FMCW devices, such as 24 GHz, 76–81 GHz, or sub-tera-Hertz, breathing-related centimeter-range displacements manifest in negligible IF changes, yet large phase changes, which can easily exceed the value of 2π, cause the phase-wrapping effect.

The phase-wrapping phenomenon, combined with the complex structure of the reflective surface patches of the human body and breathing-unrelated motion, makes accurate vital activity monitoring difficult. Therefore, recently reported approaches to human activity recognition using FMCW radar-based monitoring are due to the implementation of computationally intense algorithms that typically employ Discrete Fourier Transform (DFT) for object representation extraction followed by the application of deep neural network classifiers. Among FMCW radar applications unrelated to vital activity analysis, impressive results regarding person identification [18], posture tracking [19,20], pedestrian detection [21], or gesture recognition [22,23] have been achieved. The applications of such radars are not limited to human targets, as they have also found use in precision farming [24].

FMCW radars are currently intensely researched as a vital activity detection and monitoring technology, although other radar-based approaches have also been successfully implemented, such as impulse-radio ultra-wide band radars for indoor heart-rate monitoring [25]. High-accuracy breathing rate (BR) and heart rate (HR) estimation from data acquired with FMCW radar, reported in [26,27], were possible by adopting the scenario with subjects’ restricted motion (sleeping or immobilized) and by using a relatively long wavelength (24 GHz). Increasing the data preprocessing and data analysis complexity enabled more accurate BR and HR estimation, insensitive to random spontaneous gestures, as reported in [28]. Detection of the disease-specific breathing patterns in FMCW radar recordings using DFT-preprocessing and classical machine learning was proposed in [29]. Multi-person vital activity tracking was also investigated using MIMO (Multiple-Input Multiple-Output) FCMW radars combined with range gating and angular separation enabled by array antennas [6]. Person detection using FMCW has been considered in [30], where the negative class examples included solely still objects; in [31], where the negative set comprised animals; and in [32], including fans and mobile toys. For the presented methods, the datasets used are not publicly available, so the presented performances are difficult to be used as a reference for comparisons. For example, for the algorithm proposed in [32], the average AUROC was 94.36%, for [30] the average accuracy was 88%, and it varied from 68% to 94.5% between experiments, depending on the number of people in the room (best with 0, worst with 3). For [31], the sensitivity was 75.2% when considering both human and animal subjects using a single radar.

The common denominator for the majority of algorithms proposed for vital activity detection or estimation from FMCW radar-produced data is their computational complexity. Both radar-produced data representation extraction, commonly involving calculating discrete spectrum, as well as the analysis of the derived representation, often using machine learning algorithms, are computationally intense. Despite the growing interest in health monitoring infrastructures that comprise numerous intelligent sensing edge devices [1], only a few attempts have been made to design algorithms and hardware implementations to maximize the energy efficiency of the downstream task realization [33] and communication (including forthcoming 6G standards [34]). A way to attain this goal could be to depart from general-purpose CPU- or GPU-based computing in favor of task-oriented architectures, optimized for energy consumption. A custom, low-power digital VLSI processor for executing DFT-based extraction of object location-related information from FMCW radar, including beam-forming capability, has been proposed in [35]. Another energy-efficient implementation, this time based on FPGA hardware for the derivation of DFT-based representation from radar data, has been proposed in [36].

A natural choice for the classification step of the information extracted from radar-produced data is to use neural classifiers, which are the state-of-the-art machine learning methodology. As neural computations are intrinsic to analog technology, for developing energy-efficient vital activity detection devices, one can resort to algorithms that can be mapped onto analog or mixed-signal hardware (see, e.g., the methods proposed in [37] or [2]). Such computational architectures could benefit from a variety of proposals for analog VLSI implementations of different circuit techniques, physical devices [38,39,40,41], and neural models, including convolutional [42] and recurrent networks (LSTM [43], GRU [37,44]). However, it is well known that analog computing is more prone to errors (due to reduced noise margins) and inaccuracies induced by limited device matching. That is why analog circuits do not benefit as much as digital ones from the scaling of technology nodes. Therefore, only the algorithms relying on rather moderate parameter resolution (equivalent to four or six bits at most) are realistic for practical implementation.

## 2. Materials and Methods

**Figure 1:**
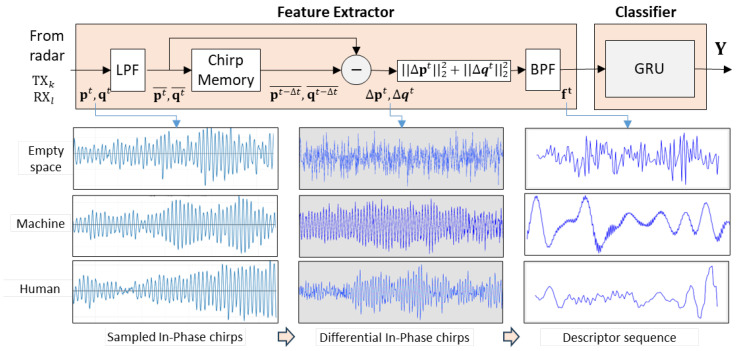
The simplified data processing pipeline for processing data produced by a single pair of transmitting–receiving antennas (TX_*k*_, RX_*l*_). LPF denotes an averaging filter, BPF is a bandpass filter tuned to the breathing frequency range; $p^{t},q^{t}$ are in-phase and quadrature vectors sampled at time instance *t*, whereas $\bar{p^{t}},\bar{q^{t}}$ are their low-pass filtering results; $\bar{p^{t-\Delta t}},\bar{q^{t-\Delta t}}$ refer to vectors sampled at t−Δt; $\Delta p^{t},\Delta q^{t}$ are differential chirps; and ${\left||.|\right|}_{2}^{2}$ denotes a squared L2 norm.

The proposed vital activity detection algorithm, schematically depicted in Figure 1, involves two modules: the feature extractor, intended for its digital implementation, and the classifier, the recurrent neural network to be implemented as an analog VLSI circuit.

An objective of the feature extractor is to summarize information on temporary object micro-displacements. Although representation learning is the solution of choice to obtain the best possible analysis outcome, such an approach would contradict the adopted architectural simplicity requirement. Therefore, to extract meaningful information from FMCW radar data, we propose to apply a descriptor that captures motion-induced energy changes in pairs of chirps separated by an appropriately chosen time interval. We postulate that the temporal evolution patterns of the proposed descriptor sequences are specific to humans and can be discriminated from patterns produced by other possible sources of motion. The produced sequences are then subject to analysis in the classifier, which is a recurrent neural network composed of gated recurrent units.

### 2.1. Descriptor Derivation

FMCW radars produce sets of vectorized data comprising the results of mixing emitted and received chirp signals (possibly by multiple transmitting and receiving antennas TX_1_ … TX_*K*_ and RX_1_ … RX_*L*_). Each signal set, originating from a sample antenna pair TX_*k*_ RX_*l*_, contains in-phase ($p^{t}$) and quadrature ($q^{t}$) *n*-element vectors, which for a discrete acquisition time instance *t*, is a mixture of harmonic components with individual elements:

(1) $$p_{j}^{t}=\sum_{i=1}^{m}A_{i}^{t}cos\left(2\pi \Delta f_{i}^{t}j+\phi_{i}^{t}\right)+N_{j}^{t},\;j=1\ldots n$$

(2) $$q_{j}^{t}=\sum_{i=1}^{m}A_{i}^{t}sin\left(2\pi \Delta f_{i}^{t}j+\phi_{i}^{t}\right)+N_{j}^{t},\;j=1\ldots n$$

where $\Delta f_{i}^{t}$ are the intermediate frequencies produced by *m* reflective surfaces, $A_{i}^{T}$ and $\phi_{i}^{T}$ are the amplitudes and phases of the harmonic components, and $N_{j}^{t}$ is the noise affecting each sample. This measurement noise is caused by several factors, such as chirp generation jitter or distortions introduced during received signal mixing. Assuming the noise to be Gaussian, one can reduce its adverse impact through simple low-pass filtering that produces in-phase vectors $\bar{p^{t}}$ and quadrature vectors $\bar{q^{t}}$ for each sampling instant *t*. Suppression of noise (the low-pass filtering block LPF in Figure 1) is the first operation in the proposed data-processing pipeline of the proposed descriptor derivation procedure.

**Figure 2:**
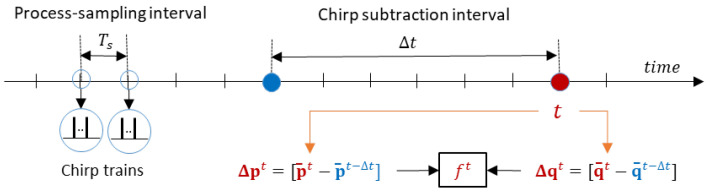
A scheme for producing differential chirps: at time intervals $T_{s}$, the radar produces short trains of chirps that are averaged and used to produce differential chirps.

The sampling frequency for monitoring breathing-related motion should be much lower than the sampling frequency used for noise-filtering. Therefore, the proposed data acquisition scheme involves the generation of short trains of chirps, originating at evenly spaced time instances separated by the interval $T_{s}$ (see Figure 2).

As each pair of filtered in-phase and quadrature vectors $\bar{p^{t}}$ and $\bar{q^{t}}$ provides a snapshot of the structure of the monitored space, information on scene object micro-displacements can be produced by subtracting vectors sampled at the time instance *t* and some preceding time instance t−Δt, where the time interval Δt is to be experimentally determined to maximize the performance of breathing activity detection:

(3) $$\Delta p^{t}=\bar{p^{t}}-\bar{p^{t-\Delta t}},\;\Delta q^{t}=\bar{q^{t}}-\bar{q^{t-\Delta t}}$$

The components of the resulting differential $\Delta p^{t}$-vectors (and analogously, $\Delta q^{t}$-vectors) can be expressed as follows:

(4) $$\begin{array}{cc} \Delta p_{j}^{t} & =\sum_{i}A_{i}^{t}cos\left(2\pi \Delta f_{i}^{t}j+\phi_{i}^{t}\right)-\sum_{i}A_{i}^{t-\Delta t}cos\left(2\pi \Delta f_{i}^{t-\Delta t}j+\phi_{i}^{t-\Delta t}\right)+{\bar{N}}_{j}^{t}-{\bar{N}}_{j}^{t-\Delta t} \end{array}$$

Assuming object displacements within the interval Δt to be small, a change in the corresponding intermediate frequencies for both chirps is negligible ($\forall_{i}\;\;\Delta f_{i}^{t}\approx \Delta f_{i}^{t-\Delta t}$). If changes in reflective patch orientations can also be neglected, we can assume that the magnitudes of the corresponding IF components for chirps to be subtracted also remain the same (i.e., $\forall_{i}\;\;A_{i}^{t}\approx A_{i}^{t-\Delta t}$). With these assumptions, it follows that differential chirps $\Delta p^{t}$ and $\Delta q^{t}$ are the sums of periodic components of magnitudes modulated by factors proportional to phase differences, with individual components of the following form:

(5) $$\Delta p_{j}^{t}=-2\sum_{i=1}^{m}A_{i}sin\frac{\Delta \phi_{i}^{t}}{2}\cdot sin\left(2\pi \Delta f_{i}^{t}j+\frac{\phi_{i}^{t}+\phi_{i}^{t-\Delta t}}{2}\right)$$

for the $\Delta p^{t}$ vector, and:

(6) $$\Delta q_{j}^{t}=2\sum_{i=1}^{m}A_{i}sin\frac{\Delta \phi_{i}^{t}}{2}\cdot cos\left(2\pi \Delta f_{i}^{t}j+\frac{\phi_{i}^{t}+\phi_{i}^{t-\Delta t}}{2}\right)$$

for the the $\Delta q^{t}$ vector, where $\Delta \phi_{i}^{t}=\phi_{i}^{t}-\phi_{i}^{t+\Delta t}$ denotes the phase difference.

The expressions (5) and (6) comprise sets of complementary harmonics scaled by identical, phase-difference-dependent factors. Therefore, a reasonable quantitative descriptor of the amount of motion that occurs between chirps separated by the interval Δt could be the aggregation of squared components of the vectors $\Delta p^{t}$ and $\Delta q^{t}$, which approximately reflects the mean energy of differential chirps:

(7) $$f^{t}=\frac{1}{n}\sum_{j=1}^{n}\left({\left(\Delta p_{j}^{t}\right)}^{2}+{\left(\Delta q_{j}^{t}\right)}^{2}\right)$$

Note that the proposed descriptor is nonlinear (through the sine function) with respect to displacements that induce phase differences in individual harmonics. In addition, displacements that exceed the radar wavelength cause random jumps in corresponding harmonic contributions. However, the proposed aggregation of components (7) enables partial compensation of the adverse phase-wrapping effects caused by the excessive displacements of various reflective patches of complex objects. FMCW radars are typically equipped with multiple transmitting and receiving antennas, enabling the recording of the same reflected waves but with different phase shifts. Using different combinations of input–output antennas (different channels) is another source of information that can prove beneficial in reducing phase-wrapping effects. If more than a single input–output channel is used, the descriptor (7) becomes a vector with as many individual components as the number of channels considered.

The proposed descriptor provides an estimate of the displacements that occur within the time interval Δt. The procedure of its derivation is extremely simple and can be easily implemented using a custom digital architecture that comprises a memory and elementary arithmetic units. A sequence of descriptors generated for subsequent sampling intervals (see Figure 1) is expected to match a pattern characteristic to breathing-related motion if humans are present within the monitored space and to be different from patterns produced by other moving objects such as fans, curtains, or robotic devices. As the considered patterns are of an intricate nature, with large within-class scatter and between-class similarities, to derive the vital activity detector, one needs to employ machine learning and use training examples to build an appropriate problem–solution framework.

### 2.2. Sequence Classifier

The two main reasons for selecting neural networks for the classification of descriptor sequences (scalar if only a single data acquisition channel is used or vectorized for multiple channels) are their capability to handle difficult data analysis tasks and the feasibility of their analog VLSI implementation. The analog VLSI technology offers a framework for implementing neural networks that is attractive, energy-efficient, and easy to integrate with digital programming [38,45], provided that their architectures are relatively simple and that the critical technology-related design constraints are met.

Viable candidates to cope with the posed, hard sequence analysis task involving intricate temporal patterns, nonlinear phase-displacement relationships, multi-path signal propagation, and interferences caused by the presence of different moving objects, are recurrent neural networks (RNNs) or transformers. Unfortunately, the transformers are infeasible for implementation in analog VLSI for the considered task due to the fundamental infeasibility of long-term analog storage of data required by its temporal attention layer. This makes RNNs the only valid candidates for the classifier computational architecture. However, since long sequences are subject to analysis, vanilla RNNs cannot be used, and one needs to resort to models that preserve long-term data relationships, such as the Long Short-Term Memory (LSTM) network [46] or the Gated Recurrent Unit (GRU) network [14]. LSTM cells are quite complex, which makes analog VLSI LSTM implementation problematic. On the contrary, GRU networks have been proven to provide performance comparable to LSTM networks, and they have already been implemented in analog VLSI [37], so they have been chosen as a sequence classification means for the proposed architecture. To verify the feasibility of such a network realization as well as to assess constraints induced by analog hardware, some critical function blocks have been designed, implemented in silicon, and tested through measurements.

#### VLSI Implementation of Classifier Components

Since the analog structures do not benefit from technology scaling as much as digital designs, a relatively inexpensive process (0.35 μm CMOS node from X-Fab, Erfurt, Germany) was selected for cost-efficient fabrication. This process includes thicker oxide transistors, which can withstand higher voltages. Therefore, it offers an opportunity to test a design based on energy-efficient floating-gate MOSFET (FG-MOS) structures [45,47], and even a custom design of quasi-analog non-volatile memories.

**Figure 3:**
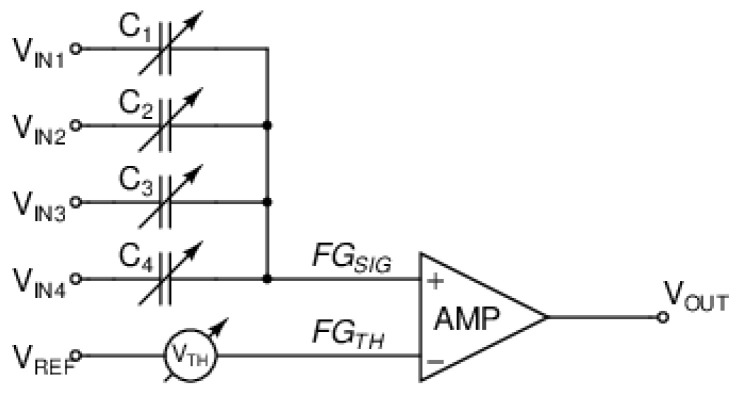
Neuron circuit based on a floating-gate differential input amplifier with programmable capacitive synapses and adjustable threshold.

A simplified schematic of a neuron, which provides a summation of multiple weighted inputs and a nonlinear output activation whenever the threshold is exceeded, is shown in Figure 3. For testing purposes, the neuron is equipped with four inputs, to which individually programmable (4 bits and sign) synaptic weights are applied. The neuron is based on an amplifier, which provides the output nonlinearity and threshold, whereas the synapses are based on capacitor banks with switches.

**Figure 4:**
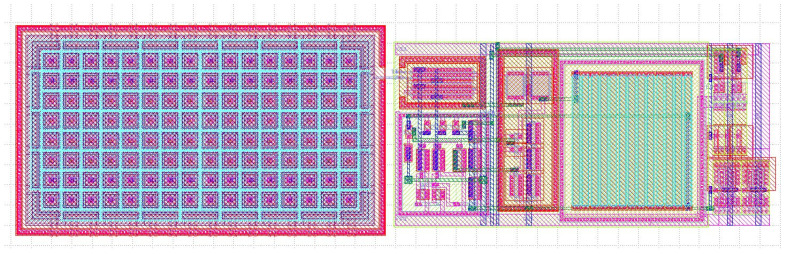
Layout of a 4-input neuron from Figure 3. The left half of the layout is occupied by the capacitor bank implementing 4 synapses with 4-bit and sign weights. The total silicon area is 135μm × 45μm.

The layout of a single neuron is shown in Figure 4. The left half of the layout is occupied by a bank of 128 unit capacitors, which allow for the routing of either eight synapses with unsigned 4-bit programmability or four synapses with 4-bit and sign programmability. For a larger number of inputs with 4-bit and sign programmability, a larger capacitor bank is needed. However, one should keep in mind that increasing the number of inputs with maximal programmability will inevitably lead to decreased sensitivity of the system to the least significant bits (LSBs), as their contribution to the overall charge sharing capacitance becomes smaller and smaller. From our experience, up to eight inputs with 4-bit and sign programmability should be possible, assuming the theoretical extreme cases, in which all synapse weights are programmed to their positive (or negative) maximum values, are rare and/or irrelevant. However, keeping a number of inputs in the range of 4–6 is more practical.

Each input signal goes through programmable capacitive coupling (synaptic weight), determining the strength with which it affects the neuron. The computation is performed in an analog way by charge sharing on the floating-gate input node, $FG_{SIG}$, of the amplifier, AMP. Such an approach allows for highly energy-efficient computation as a minimal current of charge redistribution flows only during the moment of computation.

**Figure 5:**
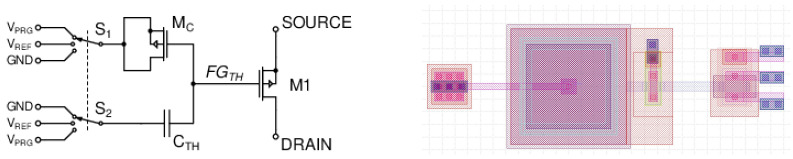
Test structure for adjustment of a neuron threshold. Floating-gate node works as analog memory storing the threshold coefficient. The layout occupies a silicon area of 28μm × 9μm.

The adjustment of the amplifier reference threshold also relies on a floating-gate structure, which allows for the setting and non-volatile storage of the tuning coefficient. The designed structure and its layout are shown in Figure 5. Synchronously operating switches $S_{1}$ and $S_{2}$ allows for reversing the polarity of the programming voltage, $V_{PRG}$, to charge or discharge the floating-gate node, $FG_{TH}$. In this way, it is possible to increase or decrease the potential of the amplifier’s negative input and thus tune the neuron’s threshold. Variable charge storage on the $FG_{TH}$ node effectively resembles an analog non-volatile memory (NVM). During the computation, both switches connect to a global reference voltage, $V_{REF}=1.65$ V, set equal to half of the supply voltage.

**Figure 6:**
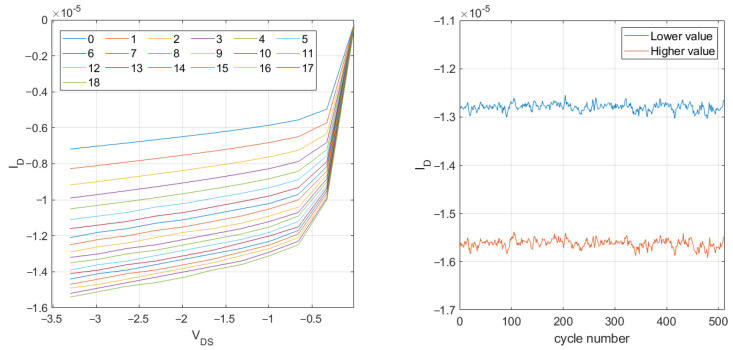
Analog memory programming with the application of subsequent pulses (**left**) and programming repeatability test revealing good, <2% inaccuracies in output current (**right**).

To assess the programmability range, the accuracy, and the stability of the analog memory, multiple tests were conducted. First, the switches $S_{1}$ and $S_{2}$ were connected to a reference voltage, $V_{REF}=1.65$ V, and the output characteristic of the MOS transistor was measured by sweeping the drain-source voltage, $V_{DS}$, from 0 to 3.3 volts in ten steps and measuring the drain current at each step. Then, a series of 6-volt programming pulses were applied in one direction. After each programming pulse, the input was connected to $V_{REF}$, and the output characteristic was measured again. Typical results from these tests are shown in the upper part of Figure 6. Similar results were obtained with programming pulses of opposite polarity, which gradually reduced the current value. As can be observed, the changes in the current value are becoming smaller with each following programming pulse. This is natural when voltage pulses of equal amplitude and duration are used because the change in the potential at the node $FG_{TH}$ after each pulse affects the tunneling current and thus the amount of charge transferred during the next programming pulse. This effect can be mitigated by performing the programming either in a closed feedback loop or by using a variable number of more granular pulses for each desired change. Nevertheless, the tuning range obtained in these tests shows that 16 distinguishable values, i.e., 4-bit programmability, can be realized in this way.

In another test, switching between two arbitrarily selected current levels was tested over 512 cycles. As can be seen in the lower part of Figure 6, the programming of the floating gate yields stable drain currents with value variations of less than 2%. The lower current value is 12.8μA (±0.25μA), whereas the higher current value is 15.6μA (±0.27μA).

As a result of the performed measurements, the following major design constraints have been identified for the derivation of neural vital activity-detecting networks. The first one is concerned with weight representation accuracy, which is limited to four-bits plus sign only, as this resolution provides correct weight programming. The second one is concerned with the size of the weight vector, which should be at most 6, to ensure correct dot product calculations. Moreover, it is challenging to implement long-term analog memories in silicon in an efficient way; thus, neural architectures that implicitly require the presence of such storage should be avoided.

## 3. Results

An experimental evaluation of the proposed algorithm was performed using a two-category dataset of indoor FMCW radar recordings (Table 1). The first category of recordings involved monitoring closed spaces in the presence of humans. The resulting ‘Humans’ class comprised recordings in which a single person or multiple persons were present in the monitored space, being either in the radar’s line of sight or optically hidden. The second category, the ‘No humans’ class, involved scenarios where the space was empty or comprised of moving objects. These objects included randomly moving curtains and two types of mechanical devices: rotating fans and a robotic arm performing reciprocal motion. The devices’ motion parameters were a match to breathing cycle intervals and ranges. The selected, real-world disturbers have the ability to generate motion patterns mimic human presence, addressing a gap in publicly available datasets while aligning with recent research frameworks emphasizing more realistic detection challenges [32].

The dataset recordings pose severe challenges to the proposed approach. The intrinsic complexity of descriptor patterns, originating from complex, large object motion and phase-wrapping effects, is further increased by additional phenomena that disrupt data acquisition: multi-path signal propagation in indoor environments and interferences caused by the presence of multiple breathing subjects. DFT-based algorithms offer methods (e.g., spatial binning) for coping with these extra factors. The objective of the evaluation was to find out whether GRU networks can successfully learn to discriminate the temporal patterns of the proposed simple motion descriptors.

The recordings were taken using a Texas Instruments IWR1443 BOOST FMCW radar board operating at 77–82 GHz, interfaced using a DCA1000EVM data acquisition card. The radar generated 175 μs-long chirps with linear frequency modulation at a rate of 20 MHz/μs. Each recording comprised scans taken at a 1 kHz sampling frequency, where each scan was composed of twelve sets of 512-element floating-point vectors, generated by twelve different configurations of three receiving (RX_1_…RX_3_) and four emitting (TX_1_…TX_4_) antennas. Each resulting set comprised a pair of the in-phase and quadrature vectors.

### 3.1. Experimental Scenarios

Multiple experimental scenarios for different combinations of the proposed procedure hyperparameters were considered. The first category of hyperparameters was the number of input data channels: all 12 pairs of vectors per scan (one for each antenna combination TX_*i*_-RX_*j*_), three pairs of vectors per scan (from the channels TX_1_-RX_3_, TX_4_-RX_1_, and TX_2_-RX_2_), and only a single pair of vectors per scan (from the combination TX_1_-RX_1_) were used as the input to the algorithm. The first scenario provides an abundance of information that can be used to cope with phase wrapping errors, as different data acquisition configurations provide different phase offsets. A downside of this approach is the increased complexity of the data preprocessing and the resulting increase in energy consumption. In the case of the last scenario, the analysis is based only on a single ‘view’ of monitored processes provided by an arbitrarily chosen combination of receiving and transmitting antennas, which simplifies processing but may result in increased phase-wrapping vulnerability.

Three different process-observation window durations were considered—2, 6, and 12 s (a single breathing cycle is assumed to last typically between 2 and 8 s). The use of the shortest observation window was motivated by measurement convenience, whereas the longest interval was expected to provide the greatest accuracy, as it covers, on average, a couple of breathing cycles.

The last hyperparameter of the proposed algorithm is a delay between the chirps to be subtracted. The range between 100 and 400 ms was adopted as a reasonable chirp subtraction interval. The lower value was experimentally determined as the minimum time difference for observing meaningful, breathing-related motion, whereas the latter one was chosen to satisfy (with a safety margin) the Nyquist condition for the maximum assumed breathing rate, which is 1 Hz. Throughout the experiments, we used four values for the delay: Δt=100,Δt=150,Δt=250, and Δt=400 ms.

The training and test sets were disjoint, and observation sequences were extracted from dataset recordings by splitting the original sequence into non-overlapping segments of length equal to the observation window duration. This produced training datasets comprising approximately twelve-thousand samples (for 2 s observation windows), over four-thousand samples (for 6 s windows), and over two-thousand samples (for 12 s windows).

### 3.2. Feature Extraction Procedure

When no moving objects are present in the radar-monitored space, scans collected at subsequent sampling intervals should contain identical information. However, due to imperfections in the data acquisition system, such as distortions during chirp generation, noisy chirp mixing, and noise of the transmitting and receiving channels, the collected data become noisy. As different possible sources of acquisition noise can be considered independent, a plausible assumption is that they have a Gaussian distribution. To verify this hypothesis, a set of Shapiro–Wilk tests for normality was performed on randomly sampled fragments of recordings taken for different indoor environments. For the null hypothesis that the observed variations in chirp magnitudes are Gaussian, we obtained *p*-values in the range of 0.6–0.8, which clearly gives no evidence for its rejection. As a consequence, to reduce the noise, a simple low-pass filtering step was adopted (the module ‘LPF’ in Figure 1), where the mean value of the three consecutive scans, comprising pairs of p and q vectors, is calculated.

As presented in Figure 1, the memorized filtered scanning results produced by the radar at a time instance t−Δt are subtracted element-wise from the corresponding results produced at the current time instance *t*. Depending on the adopted data acquisition scenario, subtraction involves two sets comprising 12 pairs, 3 pairs, or a single pair of vectors p and q. For each pair of differential vectors Δp and Δq, the energy estimate is calculated according to (7), producing either a sequence of 12-dimensional, 3-dimensional, or scalar vectors. Observe that the operations involved in descriptor derivation are elementary arithmetics, so they can easily be implemented in digital hardware, such as FPGA or other custom energy-efficient designs.

### 3.3. Vital Activity Detection Results

The architecture of the analog implementation-friendly neural network for classifying descriptor sequences produced by the feature extractor was developed in two phases. As we found the severely limited weight representation resolution to be the most restrictive design constraint, we dropped it from the first phase of our experiments, which were aimed at identifying neural architectures composed of a limited number of neurons with up to six element weight vectors.

**Figure 7:**
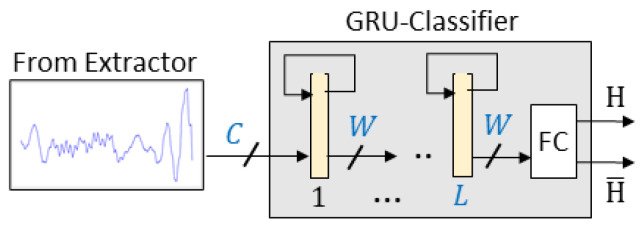
Block diagram of the classifier. The extractor-produced sequence of width *C* is analyzed by the *L*-layer GRU network (highlighted in yellow), each composed of *W*-units. Detection result: human presence (H) or absence is generated by a two-neuron fully connected layer (FC).

A schematic diagram of the tested neural models is presented in Figure 7. The network is composed of a cascade of *L* GRU layers (the values L={3,5,7,9} were considered) of the same number of units per layer (the considered values for the ‘width’ of the layer were W={6,8,16}). Descriptor sequences produced by the feature extractor and presented to the classifier were composed either of scalar values (single TX–RX combination resulting in the input dimension C=1) or vectors if multiple streams were considered (C=3 or C=12). The output of the last GRU layer was analyzed using a two-neuron fully connected layer, producing the classifier output.

Categorical cross-entropy was used for the first phase of experiments as a single-component training loss function. For each considered architecture, we executed 20 training epochs with commonly used training parameters, including the Adam optimizer [48]. In addition, optional batch normalization and dropout, with a neuron exclusion probability of 0.3, were introduced during training. All neural network models were implemented using PyTorch [49].

A modern deep neural architecture, the transformer, was also considered to provide the reference for the evaluation of the considered models. It comprised an input projection layer with GeLU nonlinearity and 32-dimensional embedding vectors, a positional encoding layer, a four-head attention layer, and three layers of densely connected neurons. As has been stated earlier, this architecture is infeasible for the considered VLSI implementation (in particular, its attention layer would require long-term analog storage of sequence elements), and it comprises too many parameters (22,370).

**Figure 8:**
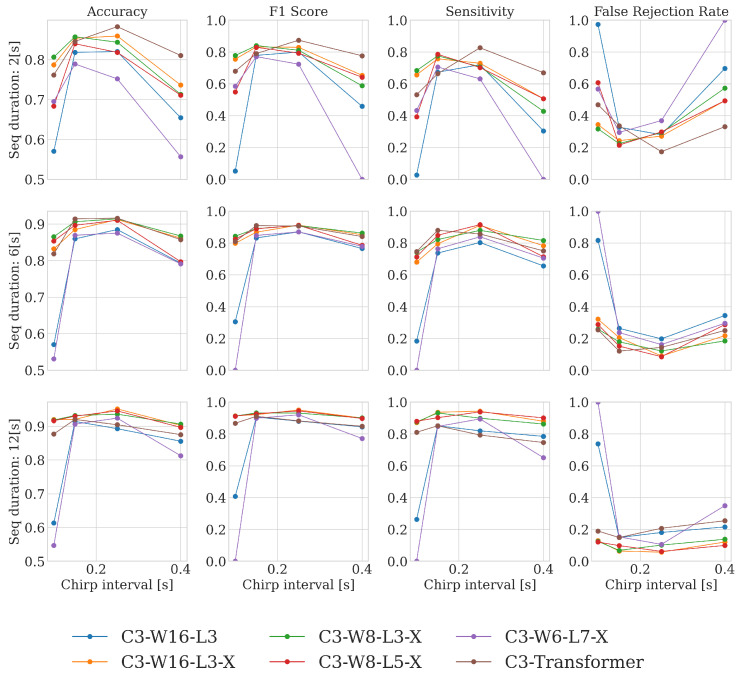
Comparative performance of six different architectures for respiratory activity detection based on the three data streams fetched from the radar (C=3) for different hyperparameter combinations: chirp subtraction interval (0.1, 0.15, 0.25, 0.4) [s] and process observation duration (2, 6, 12) [s].

**Figure 9:**
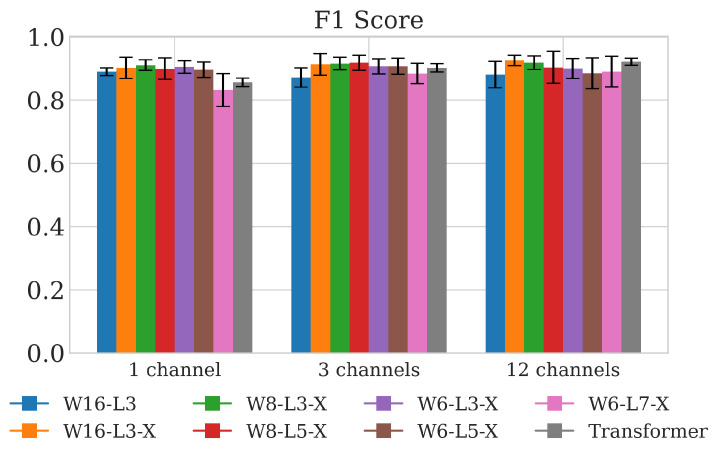
The performance of different architectures averaged over four different combinations of the method’s hyperparameters: observation window durations (6 s and 12 s) and chirp subtraction intervals (150 ms and 250 ms) for all three data-fetching scenarios.

**Figure 10:**
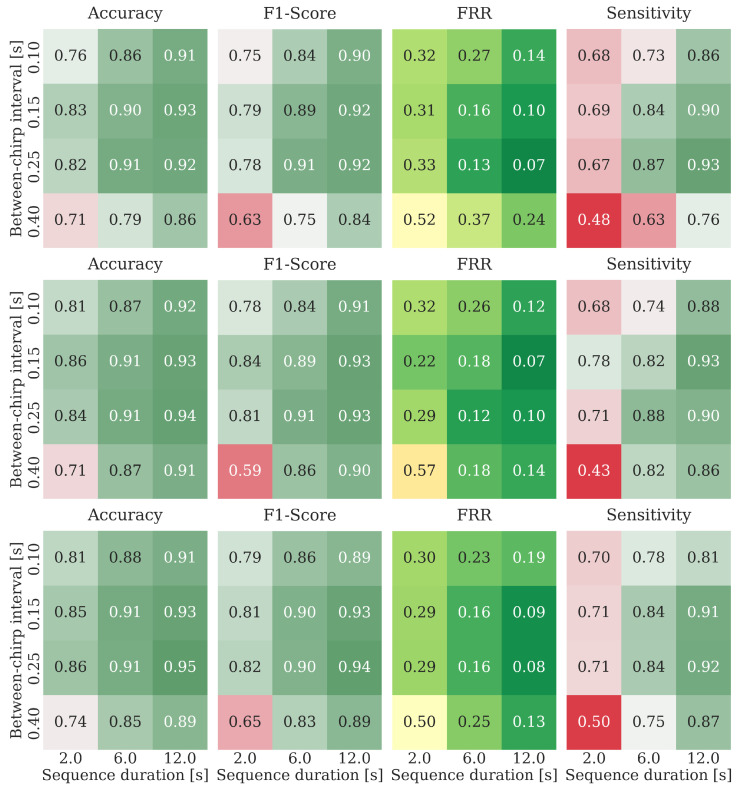
Accuracy, F1 score, False Rejection Rate (FRR), and sensitivity for different between-chirp delays and observation-window durations for the architecture W8-L3-X (3 GRU layers, each of width *W* = 8), and for the three different data-fetching modes: single-channel acquisition (**top** row), three-channel acquisition (**middle** row), and 12-channel acquisition (**bottom** row).

For each of the considered neural architectures and 36 combinations of the algorithm’s hyperparameters (four between-chirp intervals, three process observation durations, and three data acquisition configurations), the training and evaluation procedures were performed and are summarized in Figure 8, Figure 9 and Figure 10. For notation brevity, the following architecture encoding scheme was adopted: C*x*-W*y*-L*z*-X, where ‘C’ denotes the number of channels fetched from the radar-produced data, ‘W’ is the number of GRU cells per layer, ‘L’ denotes the number of GRU layers, and x,y,z are the corresponding integer values. The symbol ‘X’ indicates inclusion during the training of the optional batch normalization and the dropout.

The plots presented in Figure 8 summarize the performance of the five GRU architectures and the reference transformer for all combinations of process observation duration and between-chirp delays and for the three-channel data acquisition configuration (the results for the remaining two data acquisition configurations are very similar). The performance is evaluated in terms of the four statistical measures: accuracy, F1 score, sensitivity, and False Rejection Rate (FRR). Based on the provided results, preliminary conclusions regarding the reasonable algorithm’s hyperparameter ranges become apparent. Firstly, to correctly classify input processes, a sufficiently long process observation time is needed. This is an expected outcome, as capturing several cycles of breathing activity is clearly beneficial for decision-making. Also, there exists an optimal range of chirp subtraction delays. It seems that overly short subtraction intervals may be insufficient for capturing meaningful breathing-related motion information, whereas overly long intervals most likely increase the amount of phase-wrapping errors that distort the true motion-evolution-related contents. The specific chirp-subtraction delays that we adopt for the remaining evaluation part should be larger than 100 ms and smaller than 400 ms, and we use the six-second interval as the minimum process analysis duration. A comparative summary for selected architectures, the reduced set of hyperparameters, and all input data formats is presented in aggregated form in Figure 9, where the average performance on the four-hyperparameter set is shown.

As can be seen, the performance of the considered simple architectures is comparable to that of the transformer. Moreover, the differences among GRU architectures fed with single-channel, three-channel, and twelve-channel input are minor. This can be clearly observed in Figure 10, where the performance of the architecture comprising three layers of eight units per layer is compared among three different input data setups. The last observation is that the inclusion of input batch normalization and dropout into the training phase always proves beneficial, improving the metrics by 3–7 percent points.

It can be noted that for the proposed architectures, the problem of handling input vectors of excessive size needs to be resolved. As GRU units process both input and state, even for the 6-unit layer, each neuron of the GRU cell needs to process 12 inputs plus two biases. However, as the dot product is a linear operation, it can be distributed over a two-layered structure of linear neurons with the required input size. Therefore, all of the presented architectures are feasible for the analog implementation.

The seven network architectures that have been selected for the second phase of the experiments, together with their complexity (number of parameters), are presented in Table 2. The selection involves the four best-performing ones, the simplest and the most complex, although even in the case of the last one, the number of parameters is much lower than in the case of the reference transformer.

### 3.4. Parameter Quantization

The second phase of the proposed algorithm experimental evaluation was concerned while imposing the limited resolution constraint on the parameters of the neural model, which is necessary for the considered implementation technology. Based on preliminary simulations, a simplistic approach of quantizing the weights of the trained model to the assumed 4-bit plus sign resolution results in a catastrophic degradation of the detection accuracy.

**Figure 11:**
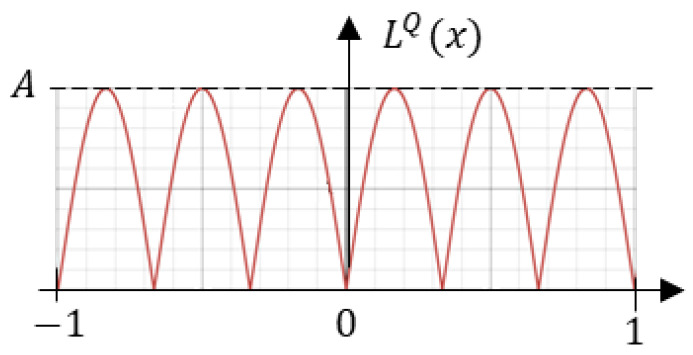
The quantization loss for the 2-bit plus sign target weight resolution. While the target architecture uses 4-bit plus sign resolution, 2-bit loss is presented here for improved readability.

To solve this problem, we propose a strategy that combines learning to solve the task using quantized weights with a simple mechanism that enables different neurons to independently learn diverse weight-quantization levels. To enforce learning of quantized parameters, we introduce the following regularization loss component (see Figure 11) that favors only the preferred parameter values:

(8) $$L^{Q}=\sum_{i}\left|sin\left(\left[B^{2}-1-\left(B\mathrm{mod}2\right)\right]\pi w_{i}\right)\right|$$

where *B* is the target weight resolution (without a sign), summation involves all parameters $w_{i}$ to be quantized, ‘mod2’ denotes the remainder modulo 2, and ‘|.|’ is an absolute value. A simple intuition behind the proposed component is to penalize weights that diverge from the minima of the periodic function, thus enforcing weights to be discrete.

For the second-phase experiments, classifier training is driven by the loss involving two components:

(9) $$L=L^{CCE}+\lambda L^{Q}$$

where λ≥0. The cross-entropy loss component ($L^{CCE}$) enforces the correct binary classification of input sequences, whereas the proposed regularization loss $L^{Q}$ pulls all weights $w_{j}^{i}$ towards the assumed quantization levels. Due to the bi-component loss structure, the resulting weights are unlikely to adopt the exact target values, so an additional ‘hard-quantization’ step is applied after the completion of learning, where all weight values are forced to the nearest quantization level.

If the quantization loss component (8) was applied directly to the neuron’s weight vectors, just a handful of the resulting admissible weight values would most likely not provide the capacity for solving complex problems. In order to alleviate this problem, we propose a simple weight-diversification method based on the application of a simple linear renormalization procedure. If we normalize elements of the weight vector $w^{i}$ of some *i*-th neuron to the values from the interval [−1, 1]:

(10) $${\hat{w}}_{j}^{i}=\frac{2w_{j}^{i}-w_{\max}^{i}-w_{\min}^{i}}{w_{\max}^{i}-w_{\min}^{i}}$$

where $w_{\max}^{i},w_{\min}^{i}$ are the extreme weight values. The dot product between the original weight vector and some input vector x can be expressed as follows:

(11) $$x^{T}w^{i}=\left(x^{T}{\hat{w}}^{i}\right)k^{i}+B^{i}$$

where the multiplier is ${\hat{k}}^{i}=\left(w_{\max}^{i}-w_{\min}^{i}\right)/2$ and the offset is ${\hat{B}}^{i}=\left(w_{\max}^{i}+w_{\min}^{i}\right)/2$.

The expression (11) reveals that if the normalized weight vectors ${\hat{w}}^{i}$ for all neurons are subject to quantization using the same, limited codebook (for each weight ‘*j*’ of a neuron ‘*i*’, ${\hat{w}}_{j}^{i}\in \left\{-1,\ldots 1\right\}$). Effectively diverse discrete weight sets, determined by neuron-specific scaling factors $k^{i}$ and offsets $B^{i}$, can be produced for different units. As a result, a few admissible quantization levels do not affect the available data processing diversity across the network. As the limited-resolution constraint also needs to be satisfied by scaling factors and offsets, during training, they are subject to hard-quantization to a set of fixed values with the adopted 4-bit plus sign accuracy.

**Figure 12:**
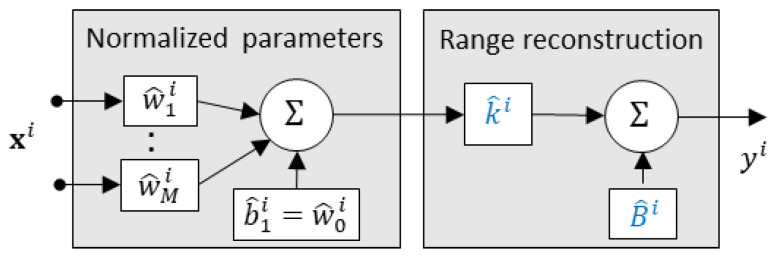
Data processing pipeline for a neuron ‘*i*’ with the quantized weights (${\hat{w}}_{j}^{i}$) and the quantized scaling and offset parameters (${\hat{k}}^{i}$ and ${\hat{B}}^{i}$).

The resulting dataflow through each of the neurons that are to be physically implemented involves the following three steps (Figure 12). First, a dot product involving inputs and quantized weights is calculated, then it is adjusted by the neuron-specific scaling factor, and finally, it is shifted using the neuron-specific offset. Scaling factors can be physically implemented by varying the gain of the underlying amplifiers, whereas offset implementation depends on the adopted signal representation mode.

**Figure 13:**
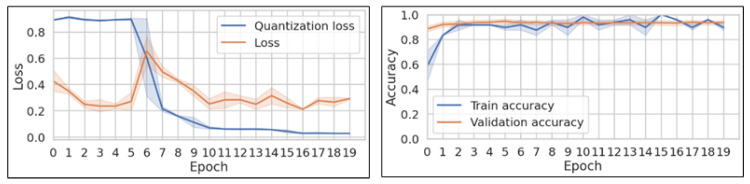
The total loss (orange) and quantization loss-only (blue) evolution during the network training, averaged per epoch (**left**), and the resulting training and validation accuracy evolution (**right**). The quantization loss is activated at the sixth training epoch.

To evaluate the proposed concepts, a subset of architectures selected for the second experimental phase (Table 2) has been trained using the proposed two-component loss (9) and evaluated. To facilitate learning, the regularization loss was activated only after the network had learned some sequence discrimination rules (as can be seen in Figure 13).

**Figure 14:**
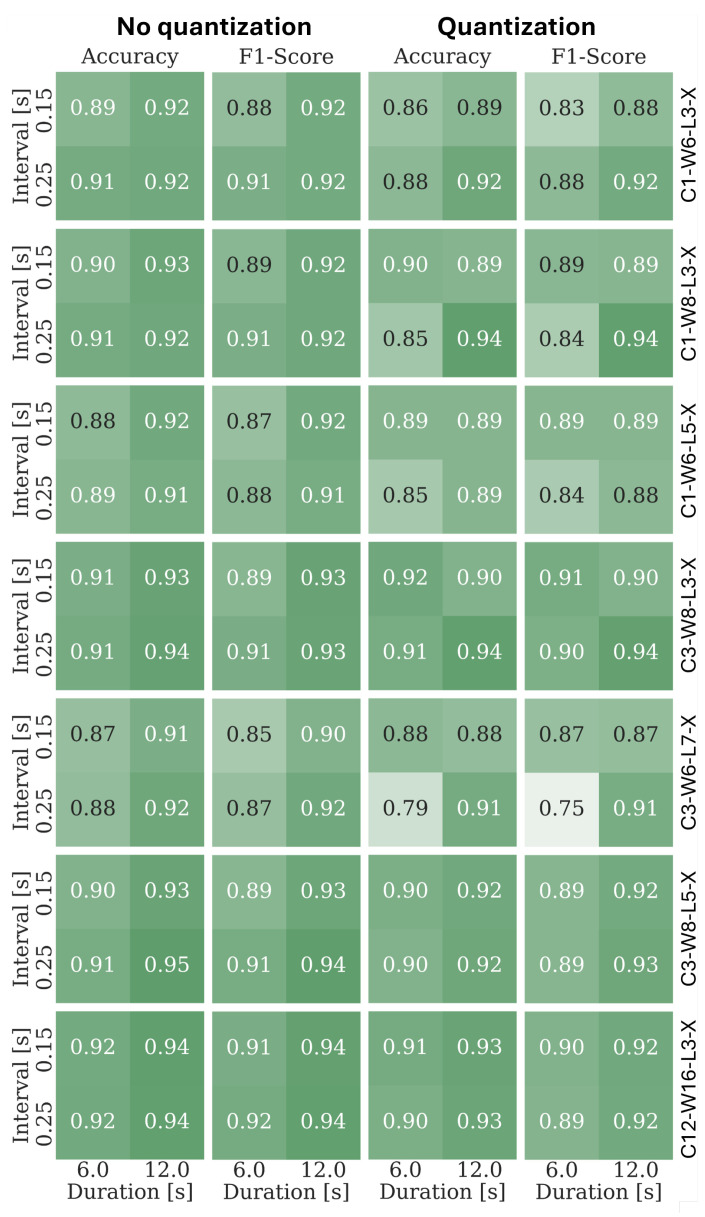
Accuracies and F1 scores of the GRU networks trained without restrictions (**left two columns**) confronted with the performance of the corresponding VLSI-feasible networks (**right two columns**). Performance for two different between-chirp delays, 0.15 s, and 0.25 s, and two process-observation windows, 6 s and 12.

For each architecture, the detection accuracies and the F1 scores are confronted with the corresponding results produced using models trained exclusively using the CCE loss (Figure 14). As can be seen, the weight quantization does not adversely affect the data analysis outcome. There are two likely reasons for retaining detection performance despite a severe reduction in weight representation accuracy. The first one can be attributed to the proposed loss component, which acts as a regularization mechanism that increases robustness to unknown sample processing. Its effects can be considered analogous to the effects of L1 regularization, with the resulting multi-level, instead of binary, parameter quantization. The second key factor that prevents performance deterioration is the proposed weight-diversification mechanism. Despite using only a single, small actual weight set, due to the proposed rescaling and offset operations (that also use quantized coefficients), the required data transformation flexibility can be retained. Also, it can be seen that the simplest architectures (C3-W8-L3-X or C1-W8-L3-X) exhibit comparable performance to the largest considered structure (C12-W16-L3-X), or even to the reference, unquantized transformer model.

The presented results show that the proposed simple approach to human detection based on radar-produced data proves viable and is well-suited for physical, digital–analog realization. The main functional module of the proposed architecture, the GRU network, despite being very simple (with a complexity of around one thousand parameters, such as C3-W8-L3-X or C1-W8-L3-X) and adapted to conform to severe VLSI implementation constraints, offers high detection performance, even after being quantized to 4-bit plus sign resolution.

## 4. Discussion

The proposed vital activity detection method, based on data fetched from the FMCW radar, is likely the simplest solution to the considered, non-trivial problem and provides a good performance. Algorithm simplicity is attained by adopting an easy-to-compute procedure for the extraction of motion-related information and by applying a frugal neural architecture. Although the proposed displacement descriptor is only moderately accurate in representing the displacements of complex objects, and the resulting descriptor temporal evolution patterns are challenging, they can be successfully analyzed using recurrent GRU-based networks. The presented scenario, where a process-representing sequence of moderate quality can still be correctly analyzed using a simple classifier, could be the method of choice for developing inexpensive, application-specific edge devices for several other real-world use-case scenarios.

We show that the proposed algorithm is well-suited for its implementation in custom mixed-signal VLSI hardware. The analog implementation feasibility of the neural module is supported by the inclusion of the constraints identified for physically realized devices in the design procedure. We show the way to overcome the inherent limitation of severely reduced parameter accuracy. Despite utilizing only a small palette of admissible weight values, we derive a neural network with diverse parameter sets that is capable of handling complex problems. As similar implementation constraints apply regardless of the adopted mode of signal representation in analog hardware, both current-mode and voltage-mode implementations of the neural models can benefit from the presented methodology.

## Figures and Tables

**Table 1:** Specifications of the dataset used in the experiments.

	‘Humans’	‘No Humans’		
		**Empty Space**	**Fan**	**Robotic Arm**
No of recordings	43	18	20	9
Total duration [s]	16,108	1310	6494	1590

**Table 2:** The parameter counts for architectures tested during the second phase of the experiments.

	C1-W6-L3-X	C1-W8-L3-X	C1-W6-L5-X	C3-W8-L3-X	C3-W6-L7-X	C3-W8-L5-X	C12-W16-L3-X
Parameters	888	1362	1368	1410	1872	2226	4602

## Data Availability

The data are available upon direct request.
